# A closed system irrigation & drainage technique for surgical evacuation of chronic subdural haematomas

**DOI:** 10.12688/f1000research.14932.1

**Published:** 2018-05-21

**Authors:** Haider Kareem, Hadie Adams

**Affiliations:** 1 Department of Neurosurgery, Charing Cross Hospital, Imperial College Healthcare NHS Trust, London, UK; 2 Department of Neurosurgery, St Mary's Hospital, Imperial College Healthcare NHS Trust, London, UK

**Keywords:** Chronic Subdural Heamatoma, Sub-periosteal drain, Close System Irrigation and Drainage (CSID), Single Burr-Hole

## Abstract

**Background: **Chronic subdural haematoma (CSDH), is a common neurosurgical disorder that is associated with morbidity and mortality affecting the ageing population. The aim is to present the treatment experience of CSDH patients treated with a technique that combines the classical single burr-hole irrigation and the continuous closed system drainage: The closed system irrigation & drainage (CSID) technique.

**Methods: **The cases undergoing CSDH evacuation with the CSID method were captured over a 4-year period at a tertiary neurosurgical centre. The authors describe the performance of this methods with respect to post-operative clinical and radiological features, including recurrence rates, complications, and length of stay.

**Results: **A total of 36 cases undergoing 42 CSID procedures (30 unilateral and 6 bilateral CSDHs) were performed, in cases ranging between 55-95 years old (median age 79 years). The rate of recurrence or significant ruminant blood in the subdural space on post-operative imaging was 11% (n=4). No cases of pneumocephalus were observed in this series (n=0). The mean (SD) skin-to-skin time for this procedure was 13.4 (4.4) minutes, with a mean (SD) length of stay of 4 (1.9) days.

**Conclusion: **We conclude that the one burr-hole closed system irrigation and drainage technique with a sub-periosteal drain seems to be a simple, effective and safe procedure for treatment of CSDH. It’s well tolerated under local anaesthesia for patients with high co-morbidities and these preliminary results indicated it may potentially be a better option for treatment of CSDH with a lower rate of post-operative complications.

## Introduction

Chronic subdural haematoma (CSDH), is a common neurosurgical disorder that is associated with high morbidity and mortality affecting the ageing population. The projected ageing of the population, and the steady increase in the use of anti-platelets and anticoagulants in modern preventive medicine, will expectedly lead to a rising incidence of anticoagulant-related complications, including CSDH ^1^. Its incidence is about (5 per 100 000 per year) in the general population, but is higher for those aged 70 years and older (58 per 100 000 per year) ^2^. The three most common described surgical techniques for subdural hematoma evacuation are: craniotomy, burr hole trephination, and twist-drill trephination ^3^. A randomised controlled trial by Santarius *et al.* in 2009 concluded that placement of a subdural drain after burr-hole evacuation of CSDH was associated with reduced recurrence rate. Recent studies reported that there are no significant differences in post-operative complication and mortality rate between the type of drain used, whether subperiosteal or subdural drain ^4– 6^. In addition to the risk of recurrence, one of the common immediate post-operative complications is pneumocephalus, CSDHs are commonly associated with cerebral atrophy and the associated increase in potential dead space in the subdural area. This factor, in addition to head positioning of the patient’s head during surgery in a neutral supine or slightly tilt, can lead to air collecting at the frontal pole of the subdural space. This can range from simple benign pneumocephalus to tension pneumocephalus with significant morbidity and mortality ^7, 8^.

We present a prospectively collected case series to present a simple surgical technique in the treatment of a common neurosurgical condition, patients presenting with a CSDH requiring surgical evacuation. This technique combines the classical burr-hole irrigation technique and the continuous closed system drainage technique by irrigating in a closed system with continues drainage: the closed system irrigation & drainage (CSID) technique. The modified irrigation technique of this approach is to minimise the operative time, risk of recurrence, and the risk of post-operative pneumocephalus. This study reflects a single surgeon’s experience in the treatment of this condition with cases treated and followed-up over a period of 4 years. We believe that this procedure has proven to be simple, effective, with a relatively short operative time and a low risk of intra- and post-operative complications. Given that the procedure can effectively be done with a single burr hole trephination, we believe that this procedure is easily tolerated under local anaesthesia which is suitable in the treatment of elderly patients with high comorbidities and those unlikely to tolerate general anaesthesia which is common amongst the population presenting with this neurosurgical disorder.

## Methods

This case series involves data collected at pre-intervention and post-intervention within routine clinical practice. The UK National Health Service National Research Ethics Service guidance on such research (National Health Service Health Research Authority, 2011) ^9^ determined that the study did not require ethical appraisal or clearance, as it was an evaluation of routine practice/National Health Service audit. Pictures and video recording of the intraoperative surgical procedure were used, alongside pre- and post-operative CT images of the same case for which informed written consent was obtained.

In this prospective cohort, we aimed to present the data of 36 consecutive patients who underwent surgery for symptomatic chronic subdural hematomas from 2014 to 2018 by a single surgeon (H.K.) at the Imperial College London NHS Trust (locations: Charing Cross Hospital and St Mary’s Hospital). Data from a total of 36 patients receiving 42 procedures are presented in this series. General patient data, including age, sex, secondary diagnosis, drug history, and other risk factors, as well as preoperative symptoms, Glasgow Coma Scale (GCS) at admission, and findings of preoperative Computed Tomography (CT) studies were collected. The side of the haematoma (right, left or bilateral), postoperative symptoms, findings of postoperative CT scan, complications, the rate of recurrence or remnant hematoma were also captured. All patients underwent single-hole trepanation, intraoperative Jacques catheter irrigation, and placement of subperiosteal closed drainage system as the first surgical treatment.

## Operative technique

**Figure 1.**
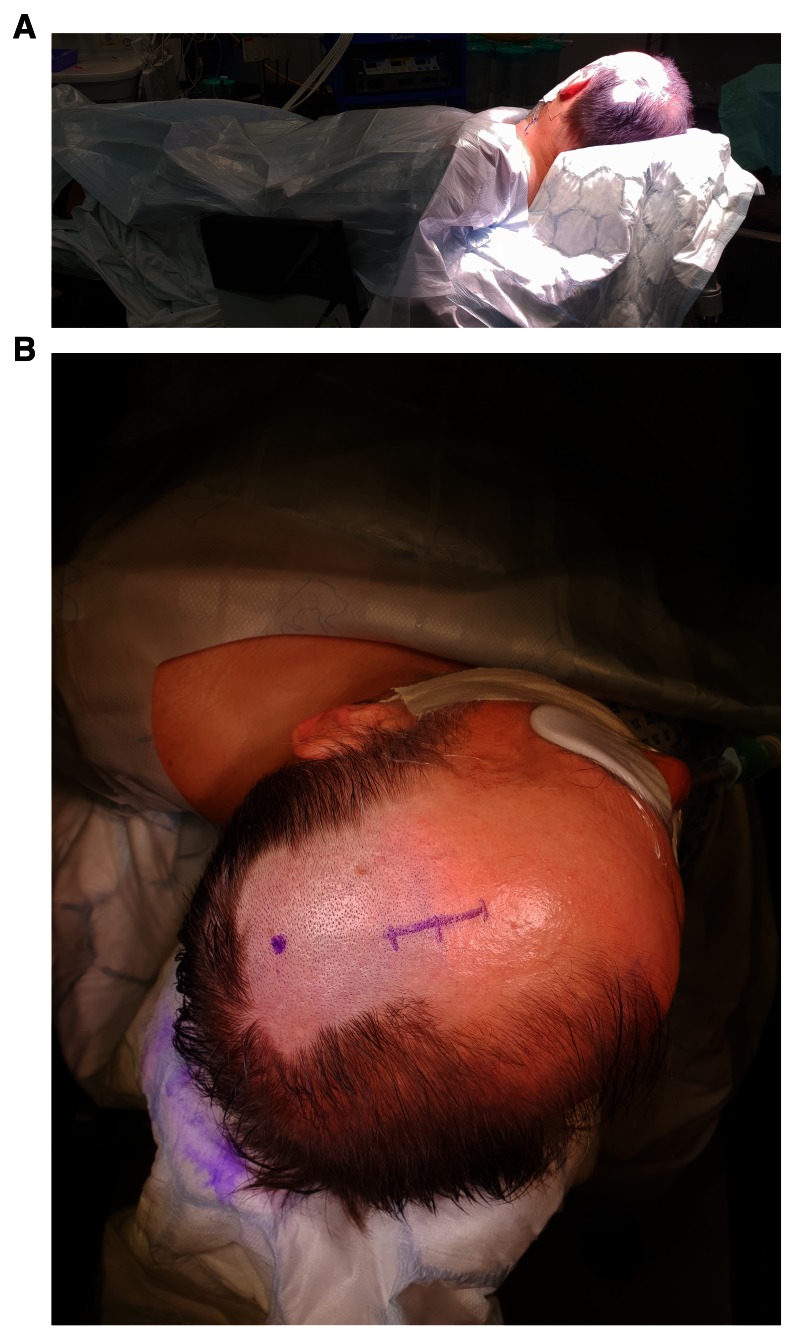
**A**) The patient is positioned slight lateral position by insertion of gel pads or sandbags under the shoulder and pelvis ipsilateral to the side of haematoma. **B**) Marking of skin incision and drain’s exit point.

**Figure 2.**
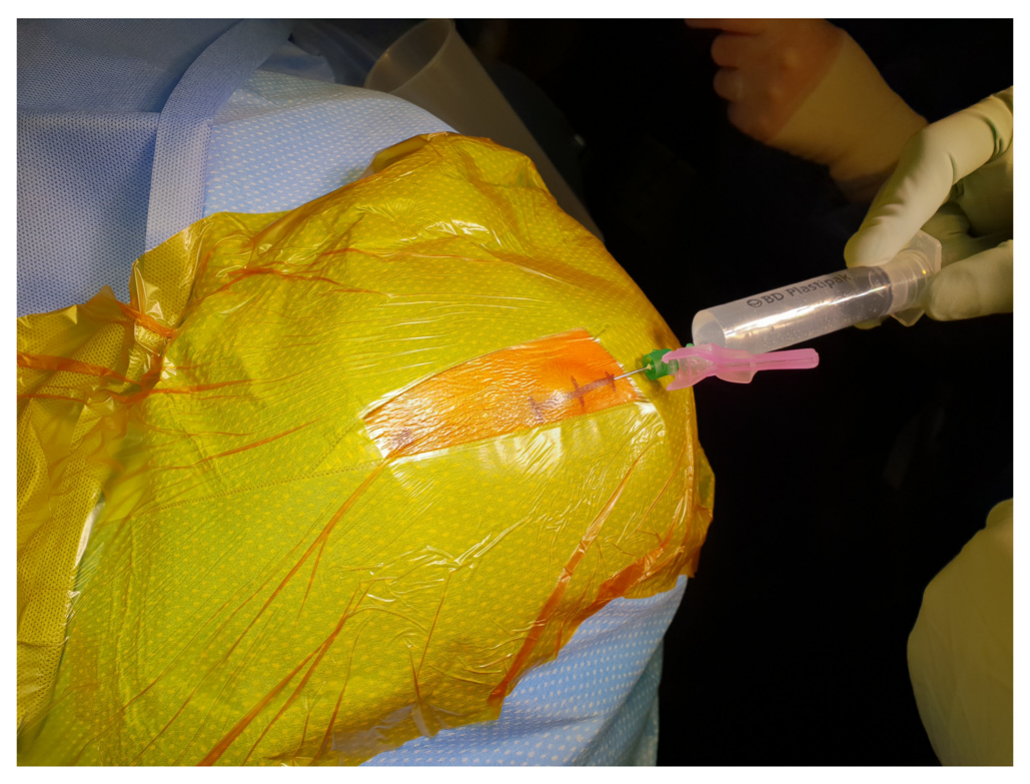
A mixed long and short-acting local anaesthetic agent is injected into the incision site, the drain exits and drain’s stitching point.

**Figure 3.**
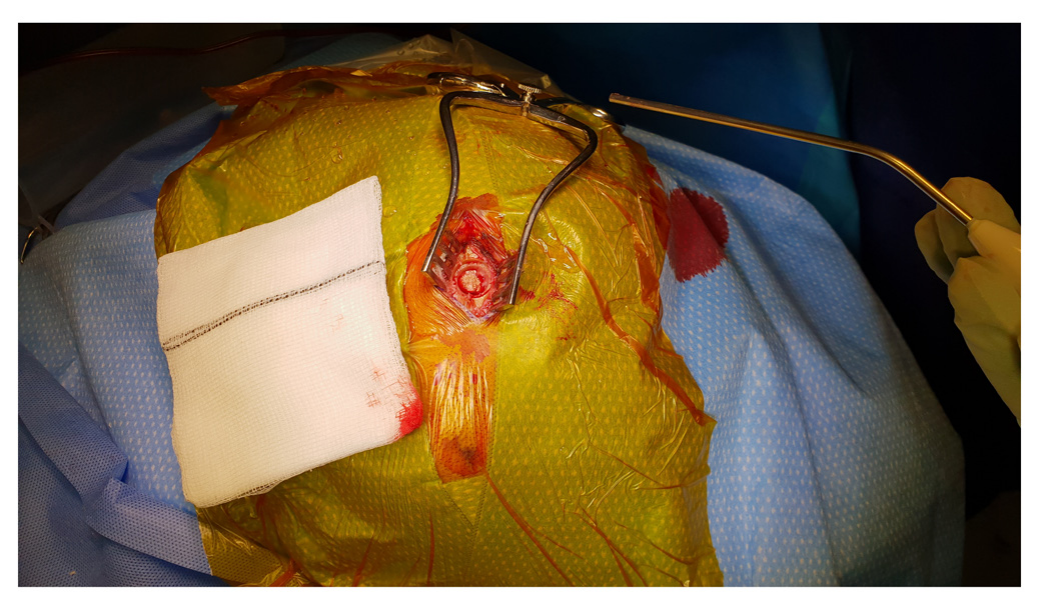
Periosteal elevation and burr hole fenestration of the skull. Prior to dural opening haemostasis of the bone edge and bipolar diathermy of the dura matter is undertaken.

**Figure 4.**
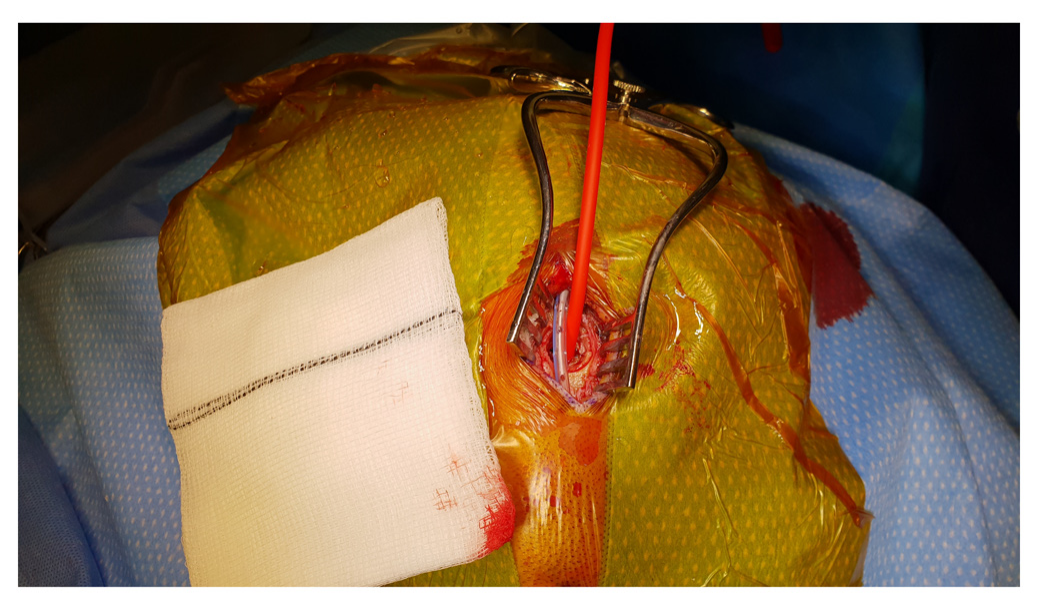
Jacques catheter and subperiosteal drain orientation at the surgical site.

**Figure 5.**
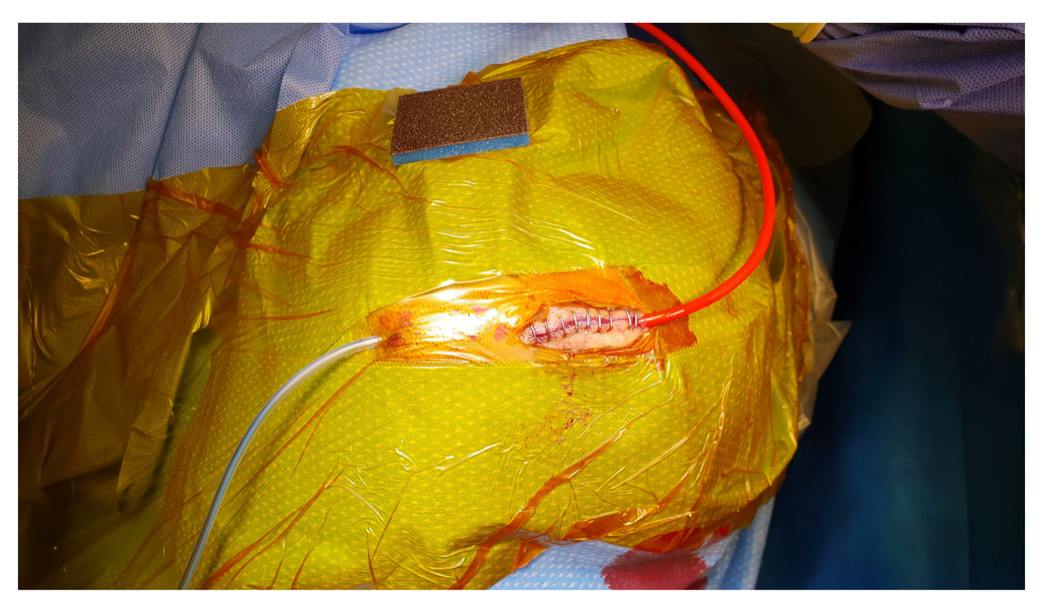
Skin closure around the Jacques catheter.

**Figure 6.**
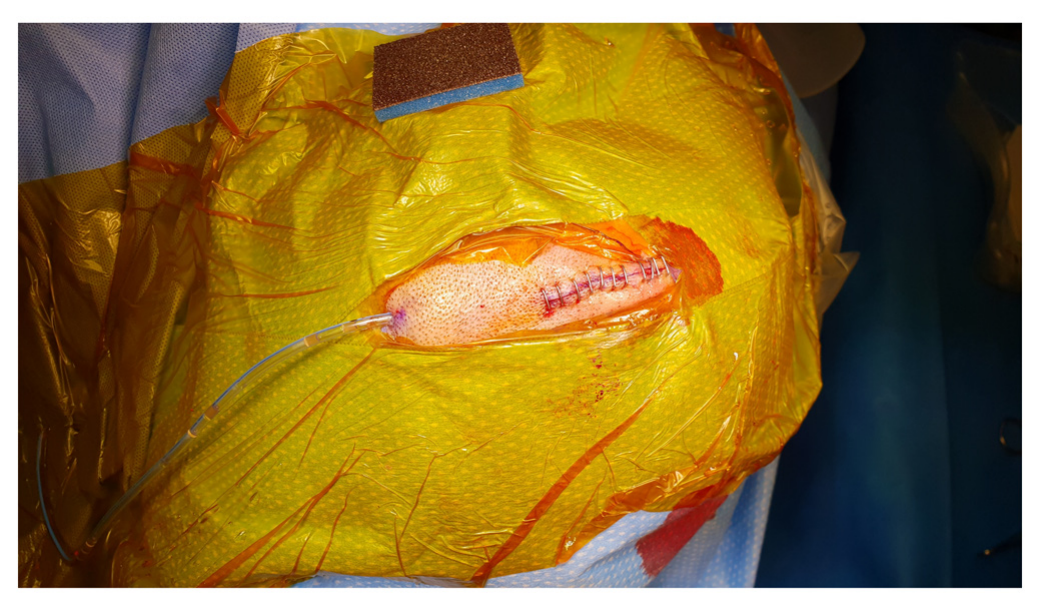
Jacques catheter removed from the wound and suture or staple of the remaining small opening in the incision.

**Figure 7.**
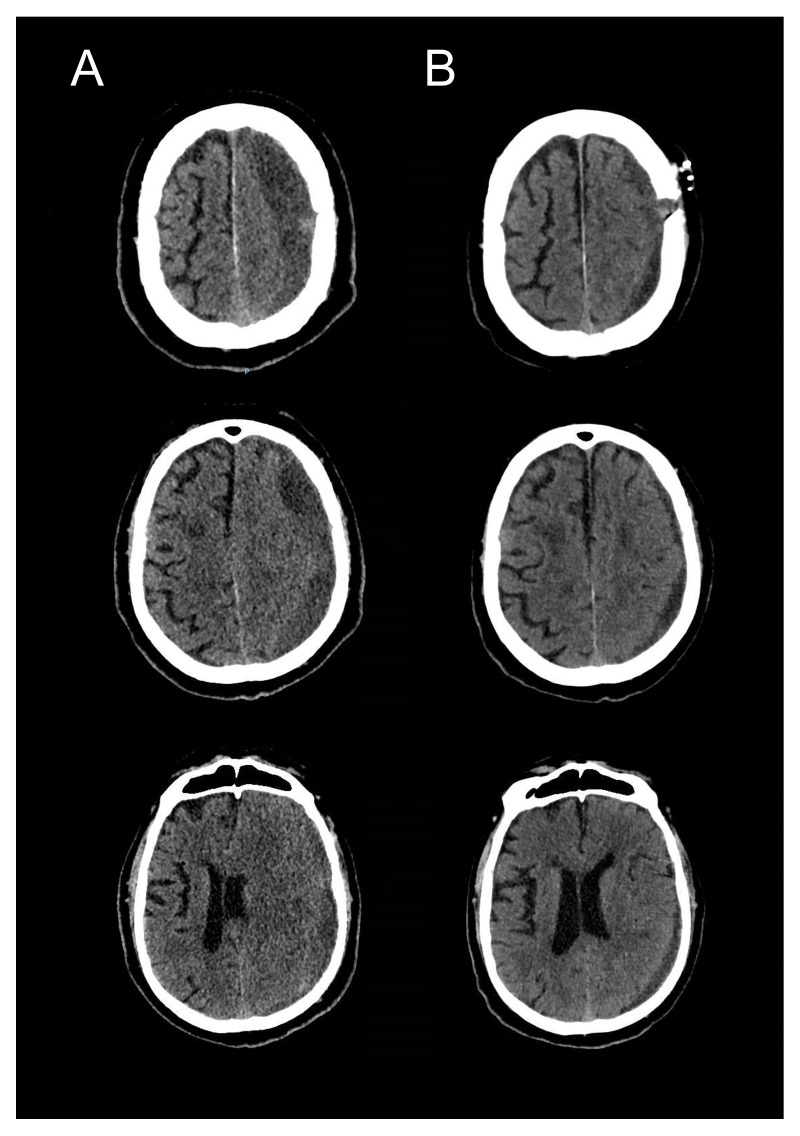
Pre- ( **A**) & post-operative ( **B**) [Day 2] cranial CT-scans demonstrating satisfactory evacuation of the subdural collection and expansion of the brain parenchyma with no pneumocephalus in the subdural space.

Prior to surgery, all patients received full clinical assessment and workup, including reversal of anticoagulation if applicable and consented/assented according to the local protocols for the procedure. One dose of prophylactic antibiotic is usually given at the time of skin incision. The patient is positioned supine or slight lateral position by insertion of gel pads or sandbags under the shoulder and pelvis ipsilateral to the side of haematoma ( Figure 1A & 1B). In case of bilateral CSDH, each side is done separately. The head of the patients is tilted to the opposite side. After skin preparation, marking the site of trepanation is at the maximum thickness of the haematoma, which should be the most superior part of the head and parallel to the floor, to avoid air entrapment in the subdural space. A mixed long and short-acting local anaesthetic agent is injected into the incision site, the drain exits and drain’s stitching point, which is usually generously infiltrated especially in the local anaesthetics cases ( Figure 2). A skin incision is made about 5cm long, followed by meticulous haemostasis of the skin edge to prevent tracking of fresh blood from the skin to the subdural space at the later stage of the procedure. Following dissection of the periosteum, a self-retaining retractor is then inserted, we usually make sure that there is no bleeding from the edges of the skin or any other point before retractor inserted. Periosteal elevation and burr hole fenestration of the skull is subsequently undertaken, haemostasis of the bone edge and bipolar diathermy of the dura matter preparing for dural opening ( Figure 3). The dura is opened in a cruciate fashion with complete cauterisation of the edges to ensure dural retraction and creating small dural defect equivalent to the osseous fenestration. At this stage, the blood starts draining from the subdural space. We make sure to fenestrate any visible membrane over the surface of the brain until the brain is visible without chasing the membrane beyond the dural defect to avoid bleeding from a remote area of the subdural space. The tip of Jacques catheter is then inserted into the subdural space for few millimetre and size 10 subperiosteal closed gravity drain inserted and tunnelled at least 5–7 cm from the surgical site. At this stage, we make sure that the fenestrated part of the sub-periosteal drain is overlying the osseous and dural defect ( Figure 4). The Jacques catheter remains at the anterior end of the surgical wound, we remove the retractor and start repairing the galea with vicryl sutures, followed by skin closure around the Jacques catheter ( Figure 5). The subdural collection then washed out with warm Ringer's lactate saline with a 50 mL syringe connected Jacques catheter. Irrigating fluid will run to the subdural space through the tip of the Jacques catheter, circulate through subdural space, irrigating all the blood and air from the subdural space and collected through the sub-periosteal drain. The process of slow irrigation continues until the outcoming irrigation fluid becomes clear. When the procedure is under local anaesthesia, we ask the patient, with anaesthetic help, to move the head from one side to another slowly which help in clearing the air and remaining blood in the subdural space. Once the irrigation fluid is clear, we pulled the Jacques catheter from the wound and suture or staple the remaining small opening in the incision ( Figure 6). The sub-periosteal drain is usually kept in for 24–48 hours to collect all the subdural ruminant fluid and blood which is further aided by the brain pulsation. In case of bilateral CSDHs, similar steps are then followed on the other side. A video of the procedure is available as part of the supplementary material ( Supplementary Video 1). Post-operative CT scan images of the same patient are demonstrated in Figure 7A and 7B.

## Results

A total of 36 patients, including 26 (72.2%) males and 10 (27.8) females underwent 42 surgical procedures (30 patients unilateral and 6 patients with bilateral trephination). The age ranges from 55–95 years old with median age 79 years (IQR 14). A total of 20 patients underwent right side surgery, 10 patient left side surgery and 6 patients underwent bilateral trephination. The vast majority, 34 (94.4%) patients had a history of trauma, mainly frequent falls. Two patients had no history of trauma reported by the patients, relatives or carer. Seven (19.4%) patients were on anticoagulation treatment, 13 (36%) patients were on antiplatelet and 4 (11%) patients were on both ( Table 1).

**Table 1.** Baseline characteristics in 36 patients treated with the CSID method.

Variables	N (%)
Total Number of patients	36
Median (IQR) Age in years	79 (14)
**Sex**, male	26 (72.2%)
**Laterality** Left-sided subdural hematoma Right-sided subdural hematoma Bilateral subdural hematoma	20 (55.5%) 10 (27.7%) 6 (16.6%)
**Clinical Presentation** Confusion Headache Hemiparesis Dysphasia Seizure	11 (30.5%) 18 (50.0%) 26 (72.0%) 1 (5.5%) 1 (2.7)
**Anti-clotting agents** On anticoagulation On antiplatelet medication	7 (19.4%) 13 (36.0%)
**Co-morbid conditions** Hypertension Diabetes Mellitus Ischaemic heart disease Dementia Arrhythmia Cerebrovascular accident Seizure Superficial wound infection	14 (38.8%) 10 (27.7%) 7 (19.5%) 2 (5.5%) 6 (16.5%) 3 (8.3%) 1 (2.7%) 1 (2.7%)
**Glasgow coma scale** 3–8 9–12 13–15	4 (11.0%) 11 (30.5%) 21 (58.3%)
**Skin to skin time in minutes (mean ± SD)**	13.4 (4.4)

 The most common clinical features on admission were hemiparesis (n=26, 72%), decreased level of consciousness with a GCS<13/15 (n=19, 52.7%), headache (n=18, 50%), confusion (n=11,30.5%), dysphasia (n=2, 5.5%) and seizures (n=1,2.7%).

All patients were re-examined post-operatively with regard to presenting symptoms. Post-operative cranial imaging (CT-scan) was done routinely 24–48 hours after the operation as a baseline investigation and also to exclude remnant haematoma or pneumocephalus ( Figure 7A & 7B), CT imaging was also performed on clinical follow-up after 1–3 months if indicated. Postoperative pneumocephalus was excluded in all patients. Symptomatic haematoma recurrence developed in 4 patients (11%), 2 of them underwent re-operation in form of mini-craniotomy with excision of the membranes in multi-layered CSDHs. The other 2 patients were treated conservatively as they were not fit for general anaesthesia. Five patients whose CT scan showed a small residual haematoma, but without any neurological deﬁcit, were followed up with satisfactory long-term resolution of the pre-operative symptoms. Postoperative complications developed in 3 patients, one patient suffered partial motor seizures that were managed successfully with anticonvulsant drugs, the two other patients suffered from pneumonia. The mean (SD) length of stay in the neurosurgical unit up until repatriation to secondary care was 4 (1.9) days ( Table 2). Further post-operative events, including complications, are summarised in Table 2.

**Table 2.** Post-operative events in 36 patients treated with the CSID methods.

Post-operative events	N (%)
**Post-operative Glasgow Coma scale** 3–8 9–12 13–15	0 (0.0%) 3 (8.3%) 33 (91.7)
**Symptomatic residual haematoma**	4 (11.0%)
**Craniotomy needed**	2 (5.5%)
**Contralateral haematoma**	0 (0.0%)
**Pneumocephalus**	0 (0.0%)
**Subdural infection**	0 (0.0%)
**Wound infection**	1 (2.7%)
**Pneumonia**	2 (5.5%)
**Deep venous thrombosis**	1 (2.7%)
**Other medical complications** Cardiac arrhythmia Severe vertigo and dizziness Pacemaker and internal cardiac defibrillator issues Severe uncontrollable hypertension	2 (5.5%) 1 (2.7%) 1 (2.7%) 1 (2.7%)
**Mortality during admission to Neurosurgical Unit**	0 (0.0%)
**Length of stay in days (mean ± SD)**	4 (1.9)

## Discussion

Although there is a wide range of opinions about the proper surgical management of this common neurosurgical condition, a scoping literature review shows no significant differences in mortality or complications rates between different surgical techniques, and complication rates of the closed draining system after subdural irrigation seems to be similar to the tradition burr-hole irrigation of the subdural space. In addition to that, there were no obvious differences in recurrence between irrigation and no irrigation. However, using a closed drain system seems to be associated with a reduction of the recurrence rate of subdural collection in compare with burr-hole evacuation only ^2, 10, 11^. In 2009, Zumofen *et al*. published the first series of patients treated with placement of a sub-periosteal drain system instead of the widely used subdural drain system after double burr-hole craniotomy for hematoma evacuation. They reported equal-to-superior results compared with previous studies regarding hematoma recurrence, mortality, and serious complications, especially postoperative seizures ^12^.

There is evidence in the literature suggest that sudden evacuation of chronic subdural hematoma may lead to growth or development of remote (contralateral) subdural, epidural or intra-cerebral haematomas ^13– 17^. The possible mechanisms behind this phenomenon include the rapid shift of the cerebral hemisphere following hematoma evacuation, over drainage of the subdural space, and cerebral hyperperfusion syndrome, although the actual pathophysiology behind this process remains unclear ^16– 20^. We suggest that the CSID technique will allow for a gradual, and physiological more natural, expansion of the brain during the irrigation and drainage process. Unlike the classical burr-hole irrigation technique, with the CSID method, the subdural collection will be slowly substituted with irrigation fluid in a closed system. Similar to the continuous closed system drainage techniques, the CSID method will also allow continues drainage without the introduction of air in the subdural space or rapid shift, however, it also offers the advantages of thorough irrigation. The cerebral pulsations will allow for a slow expansion of the brain parenchyma and a gentler expulsion of the irrigation fluid and possible remaining blood products in the subdural space through the indolent subperiosteal drain.

It has been widely described that air in the subdural space can physically block re-expansion of the brain following the evacuation of a CSDH. It has also been demonstrated that pneumocephalus increases the recurrence rate of chronic subdural haematomas, and prolongs the hospital stay and healing time ^21^. In the present cohort, no cases of (symptomatic) pneumocephalus were observed, given that post-operative symptomatic pneumocephalus was reported as relatively common complication in the literature, with 8% of the cases undergoing burr-hole irrigation procedures are followed by tension pneumocephalus ^22– 24^.

The rate of recurrence or significant ruminant blood in the subdural space on post-operative imaging was 11% (4 patients). Surgical intervention in the form of a mini-craniotomy was needed in 2 patients. Intra-operatively these cases showed multi-layered membrane adhesions to the surface of the brain. The other 2 patients with symptomatic recurrence were treated conservatively as they were not appropriate candidates for general anaesthesia to undergo a mini-craniotomy due to other significant comorbidities. We didn’t think that they would benefit from revision burr hole evacuation under local anaesthesia due to thick membrane obvious in their CT scan and failure of the first attempt to achieve the target. Reviewing the available literature, re-intervention rates after burr hole surgery with subdural drains vary between 8.3% and 30% ^3, 25– 29^, with our results falling within this range.

Post-operative infection is an uncommon complication after surgical evacuation of CSDH, with postoperative empyema being reported to occur in approximately 2% of patients using burr-hole evacuation with or without subdural drain methods ^30, 31^. We didn’t observe any deep tissue infection. One diabetic patient developed a superficial skin infection which responded well to oral antibiotic treatment.

The main limitation of this study was its small sample size. Despite including cases from a period of 4 years, the sample size of the final cohort was relatively small given it was a single centre and single surgeons experience, which can all introduce bias to our results. Whether this study lacked the adequate sample size to detect a significant difference between the presented cohort and the numbers presented in the literature or whether the results were indeed comparable remains a question. Another limitation is the inability to test risk factors that could have had an impact on recurrence or other post-operative complications. Also, it was difficult to evaluate its performance in patients with bilateral CSDHs because of the small number of patients with bilateral collections.

## Conclusion

One burr-hole closed irrigation and drainage technique with a sub-periosteal drain seems to be a simple, effective and safe procedure for treatment of CSDH. It’s well tolerated under local anaesthesia for patients with high co-morbidities and with a low rate of post-operative complication. Larger series are needed to directly compare this technique’s long-term performance to other established methods, however, these preliminary results indicated it may potentially be a better option for treatment of CSDH with a lower rate of post-operative complications.

## Data availability

Data cannot be shared because the routinely collected patient data used in the study belong to the Imperial College London and Imperial College Healthcare NHS Trust and not the authors. Researchers can apply to access the data through the Trust’s R&D Department at jrco@imperial.ac.uk.

## Supplementary material

Supplementary Video 1 - A step-by-step video demonstrating the closed system irrigation & drainage technique for surgical evacuation of chronic subdural haematomas.
