# Supplementary material for: A closed system irrigation & drainage technique for surgical evacuation of chronic subdural haematomas
Source: F1000Res. 2018 May 21;7:619. [Version 1] doi: 10.12688/f1000research.14932.1 (PMC5974591; doi:10.12688/f1000research.14932.1)
Supplement: Peer review history

# Peer review: Referee response for version 1

**DOI:** 10.5256/f1000research.16257.r34255

**Published:** 2018-05-25

**Authors:** Richard Z. Fu

**Affiliations:** 1 Department of Neurosurgery, Manchester Centre for Clinical Neurosciences, Greater Manchester, UK

This paper by authors Kareem H and Adams H presents an interesting prospective case series of patients who received an operation for the diagnosis of chronic subdural haematoma which utilised a novel yet seemingly simple, close system irrigation and drainage technique.  

 Their results show a recurrence rate not notably different from that reported in the wider literature. Their result of no pneumocephalus reported in their case series is perhaps the most interesting result. Additionally, they report a short operating time and hospital stay which might represent a potential economic benefit to the health service. 

 It would have been of value to note the clinical course of other patients in the unit who underwent a more standard operative intervention during the same time period, and for this to have been compared.

 Their paper provides a relevant and helpful review of the existing literature. Their conclusions drawn are measured and balanced, with dutiful awareness of the study's limitations.

 Their results could pave the way for a meaningful larger study to follow, where rates of recurrence, pneumocephalus and the cost benefit ratio of operating time and hospital stay can be further explored.

I have read this submission. I believe that I have an appropriate level of expertise to confirm that it is of an acceptable scientific standard.

# Peer review: Referee response for version 1

**DOI:** 10.5256/f1000research.16257.r34256

**Published:** 2018-05-23

**Authors:** Matthew A. Kirkman

**Affiliations:** 1 Victor Horsley Department of Neurosurgery, The National Hospital for Neurology and Neurosurgery, University College London Hospital NHS Foundation Trust, London, UK

This is a prospective study of 36 consecutive patients with chronic subdural haematoma presenting to a single tertiary UK neurosurgical centre over a 4-year period. The authors evaluated use of the so-called closed system irrigation and drainage (CSID) technique, finding no patients developed pneumocephalus, although recurrence or significant residual blood was seen in 11%. Skin-to-skin time was short (mean 13.4 minutes), and mean length of stay in the neurosurgical unit was 4 days.

 This is an interesting study of a common condition seen in neurosurgical practice, and a useful addition to the literature. The multimedia included add significant value to the paper. The authors discuss the limitations of their study clearly. The data suggests that this technique warrants further study in a larger population. I have a couple of questions for the authors:

  

1. Do the authors have any comparative data from patients with CSDH treated at the same centre during the same time period, but with different surgical techniques e.g. standard burr-hole irrigation? It would be interesting to compare (perhaps even statistically) surgical time, complication rates and length of stay between the different approaches. However, I do note that the authors already quote previous literature when discussing the complication rates associated with other surgical approaches to CSDH, which in itself is useful.
2. Table 1 indicates that one patient had a superficial wound infection in the “Co-morbid conditions” section – can the authors clarify exactly where this patient’s infection was, and if it was the same patient that developed a wound infection after sutrgery (as per Table 2).

I have read this submission. I believe that I have an appropriate level of expertise to confirm that it is of an acceptable scientific standard.
